# Implementation of a mobile prosthetic and orthotic care program in the VA; a qualitative study of implementation challenges and associated strategies for improvement

**DOI:** 10.3389/frhs.2024.1198191

**Published:** 2024-11-01

**Authors:** Chelsea Leonard, Jessica Young, Lauren McKown, Carolyn Klassen, George E. Kaufman, Daniel Abrahamson

**Affiliations:** ^1^ Eastern Colorado VA Healthcare System, Seattle-Denver Center of Innovation for Veteran-Centered & Value-Driven Care (COIN), Aurora, CO, United States; ^2^ VA Collaborative Evaluation Center (VACE); ^3^ Division of Health Care Policy and Research, University of Colorado Anschutz Medical Campus, Aurora, CO, United States; ^4^ VA Puget Sound Healthcare System, Seattle, WA, United States; ^5^ VISN 20 Prosthetics and Sensory Aids Services, VA Puget Sound Healthcare System, Seattle, WA, United States

**Keywords:** rural, RE-AIM evaluation framework, orthotic and prosthetic, implementation, mobile care

## Abstract

**Introduction:**

Anticipating and addressing implementation challenges is critical to ensuring success of mobile healthcare programs. Mobile Prosthetic and Orthotic (O&P) Care (MoPOC) is a new U.S. Department of Veterans Affairs (VA) program that aims to improve access to VA-based O&P services through a national network of traveling O&P clinicians who deliver care in rural communities. We conducted an iterative evaluation guided by the Reach, Effectiveness, Adoption, Implementation, and Maintenance (RE-AIM) framework to identify challenges and associated strategies for successful implementation of this mobile O&P program.

**Methods:**

MoPOC is delivered by an O&P clinician anchored at a VA medical center (VAMC). Clinicians travel to remote VA clinics and Veteran's homes with a custom vehicle which provides storage and a workshop to modify O&P devices. Each clinician is supported by a program support assistant. MoPOC was implemented in three phases. The qualitative evaluation of MoPOC implementation was conducted as part of a larger evaluation of MoPOC program outcomes. We conducted semi-structured interviews and regular check-ins with MoPOC clinicians, site managers, and stakeholders both prior to implementation and throughout the implementation process. Interviews were recorded and transcribed verbatim. Data was analyzed across sites and comparatively by phase using a rapid matrix analysis to identify themes related to adoption and implementation challenges and key strategies developed to address those challenges.

**Results:**

We identified four key themes related to successful program implementation, each with associated challenges and improvement strategies: (1) “Finding the right sites for MoPOC” through intentional recruitment and site selection; (2) Identifying the “sweet spot”: Balancing program capacity, sustainability, and MoPOC clinician satisfaction; (3) Shifting from testing to standardizing; and (4) “Being strategic with hiring” to improve program adoption.

**Discussion:**

Implementation challenges were related to recruiting and selecting successful sites, ensuring timely program adoption, balancing site level adaptation and program standardization, and scaling programs to enhance efficiency, reach, and satisfaction. An iterative approach guided by the RE-AIM framework resulted in program improvement and more rapid implementation in each successive phase. The challenges described in MoPOC implementation may be common issues in implementing new mobile programs in rural areas.

## Introduction

Mobile Orthotic and Prosthetic (O&P) care is a an increasingly common way of bringing specialty care closer to patients in the United States, as evidenced by several mobile programs serving different regions of the country (1). While Mobile O&P care has long been delivered by clinicians who occasionally visit hospitals or nursing facilities with a limited number of supplies, the O&P clinician's reliance on a workshop with specialty equipment and tools to fabricate and modify O&P devices has complicated the field's ability to deliver mobile care to patients with complex needs. Perhaps spurred by the coronavirus (COVID-19) pandemic, there has recently been an influx of programs with large specialty vehicles that deliver care at patient homes, workplaces, or primary care clinics. Research suggests that mobile healthcare successfully reaches vulnerable populations (2, 3) and increases access to specialty care services in rural or resource-limited areas (4–8). Despite the potential of mobile care to increase access to O&P services and growing utilization of mobile O&P care, best practices, common barriers, and common facilitators to implementing mobile O&P clinics are not well described in the United States (but see 9, 10). We are unaware of qualitative work that describes the challenges and considerations of implementing mobile O&P care from the perspectives of diverse stakeholders.

Understanding how to implement mobile specialty care is especially salient in the U.S. Department of Veterans Affairs (VA). On average, VA has provided O&P care to more than 300,000 distinct Veterans annually for the last decade. Though VA provides world-class O&P care, Veterans in rural areas frequently experience a variety of barriers to accessing that care. MoPOC (Mobile Prosthetic & Orthotic Care) is a national program that aims to improve access to VA O&P care through a network of mobile O&P clinicians with the ability to deliver care in a variety of more easily accessible locations including VA clinics and directly within Veterans’ homes. MoPOC services are delivered by Certified Prosthetist/Orthotists (CPOs) who travel with custom vans serving as mobile workshops. Vans are equipped with a range of equipment and the ability to provide same-day adjustments to custom prosthetic and orthotic devices.

To address the gap in the literature around implementation of mobile O&P care, we conducted an iterative qualitative evaluation of MoPOC implementation guided by the RE-AIM framework. Our goal was to describe challenges and adaptations or mitigation strategies developed to enhance the national rollout of MoPOC across the VA healthcare network.

## Methods

### Description of MoPOC

MoPOC is a Veterans Health Administration (VHA) Enterprise-Wide Initiative with funding and budgetary oversight from the VHA Office of Rural Health, programmatic oversight from the VHA Program Office of Rehabilitation and Prosthetic Services, and with program leadership organizationally aligned under Veterans Integrated Service Network (VISN) 20 Prosthetics and Sensory Aids Services. MoPOC received funding to expand in 2021 following 2 years of pilot work at VA Puget Sound Health Care System which demonstrated feasibility and effectiveness of a mobile model of care delivery to improve access to O&P VA care. The mission of the MoPOC program is to ensure reasonable access to VA clinical O&P services for all Veterans by reducing or eliminating travel burden. There are currently 96 VA medical centers (VAMC) across the enterprise that offer traditional O&P clinical services, most of which are in large metropolitan areas. As MoPOC is an Office of Rural Health program, special emphasis is placed on meeting the needs of Veterans living in rural communities. To accomplish this, each MoPOC clinical care provider is equipped with a custom-modified vehicle in which they travel to see Veterans at VA CBOCs, which are small VA clinics typically located in rural areas. CBOCs usually offer only primary care services, and the integration of MoPOC into CBOCs expands access to care by broadening the reach of O&P clinical staff anchored out of urban VAMCs. The MoPOC vehicle is not a Mobile Medical Unit; it provides storage and a light-duty workshop in which the orthotist-prosthetist can make adjustment to O&P devices. When Veterans are unable to seek care at CBOCs due to the nature of their disability, they may also be seen at home.

**Figure 1:**
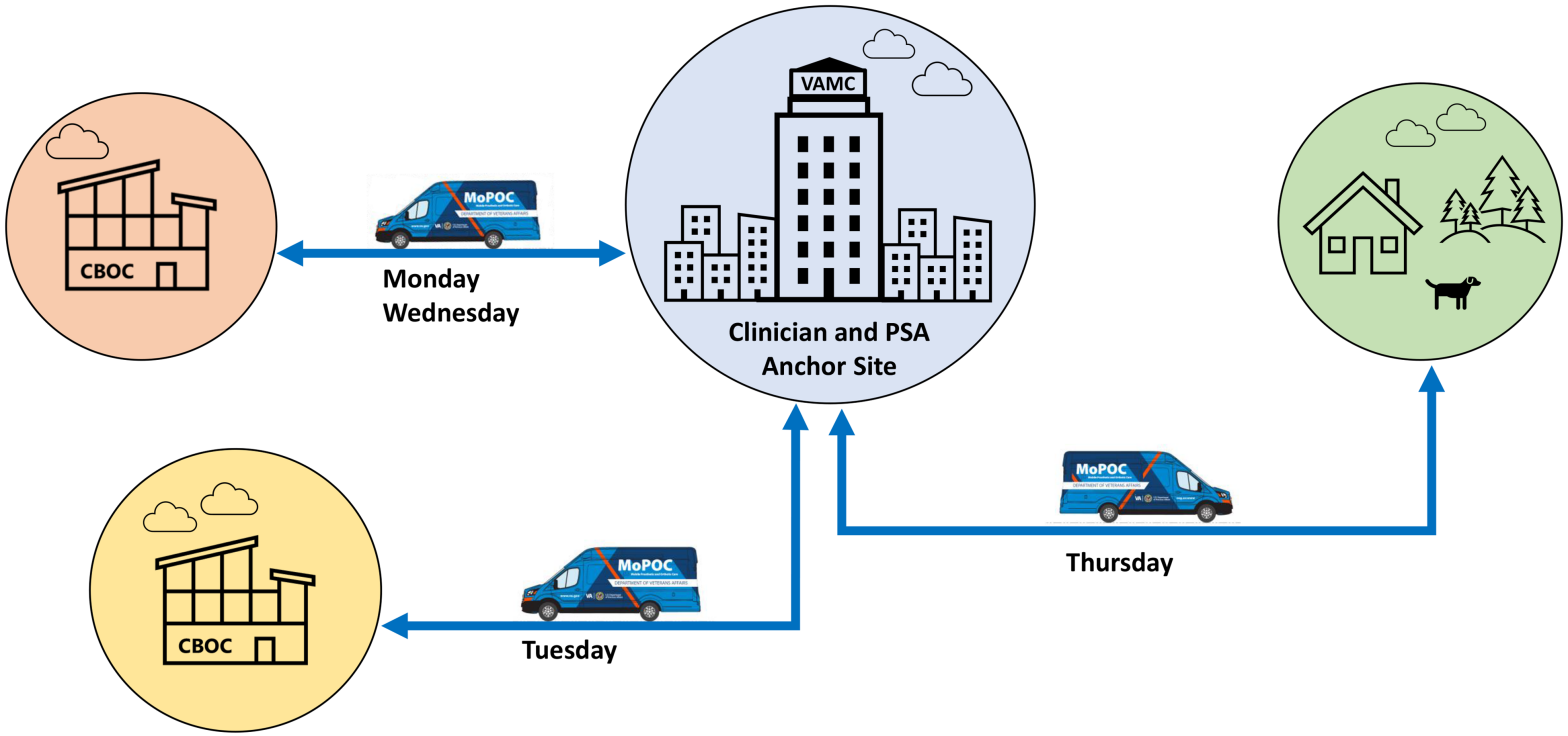
MoPOC care model. MoPOC services are anchored from a VA Medical Center (VAMC). The Certified Prosthetist/Orthotist (CPO) and Program Support Assistant (PSA) are co-located at the VAMC. The CPO travels from the VAMC to two to three Community Based Outpatient Clinics (CBOCs) and patient homes to deliver care in convenient locations for rural Veterans. The PSA works with the CPO on administrative tasks like ordering items and following up with patients, and helps problem solve scheduling issues that may arise.

MoPOC clinical staff are anchored out of a VAMC (anchor site) that provides O&P care and is fully equipped with a brick-and-mortar fabrication laboratory. Implementation of the program at each site is led by a local MoPOC Site Manager under guidance from the MoPOC Program Office. The site manager is responsible for hiring local staff and working with the Program Office team to define the scope of MoPOC at the site and guide MoPOC clinicians and staff through the implementation process. Each clinician is supported by a local full time Program Support Assistant (PSA), who manages communications with Veterans and referring providers, works with dedicated scheduling staff to coordinate the clinician schedule, assists in route planning, and facilitates equipment and supply orders. MoPOC clinicians do not engage in overnight travel and are expected to conduct 4–6 patient encounters during each of the 3 weekdays they travel to CBOCs, thus the distance to each CBOC is limited to about 100 miles or less. Figure 1 illustrates the MoPOC care model.

**Figure 2:**
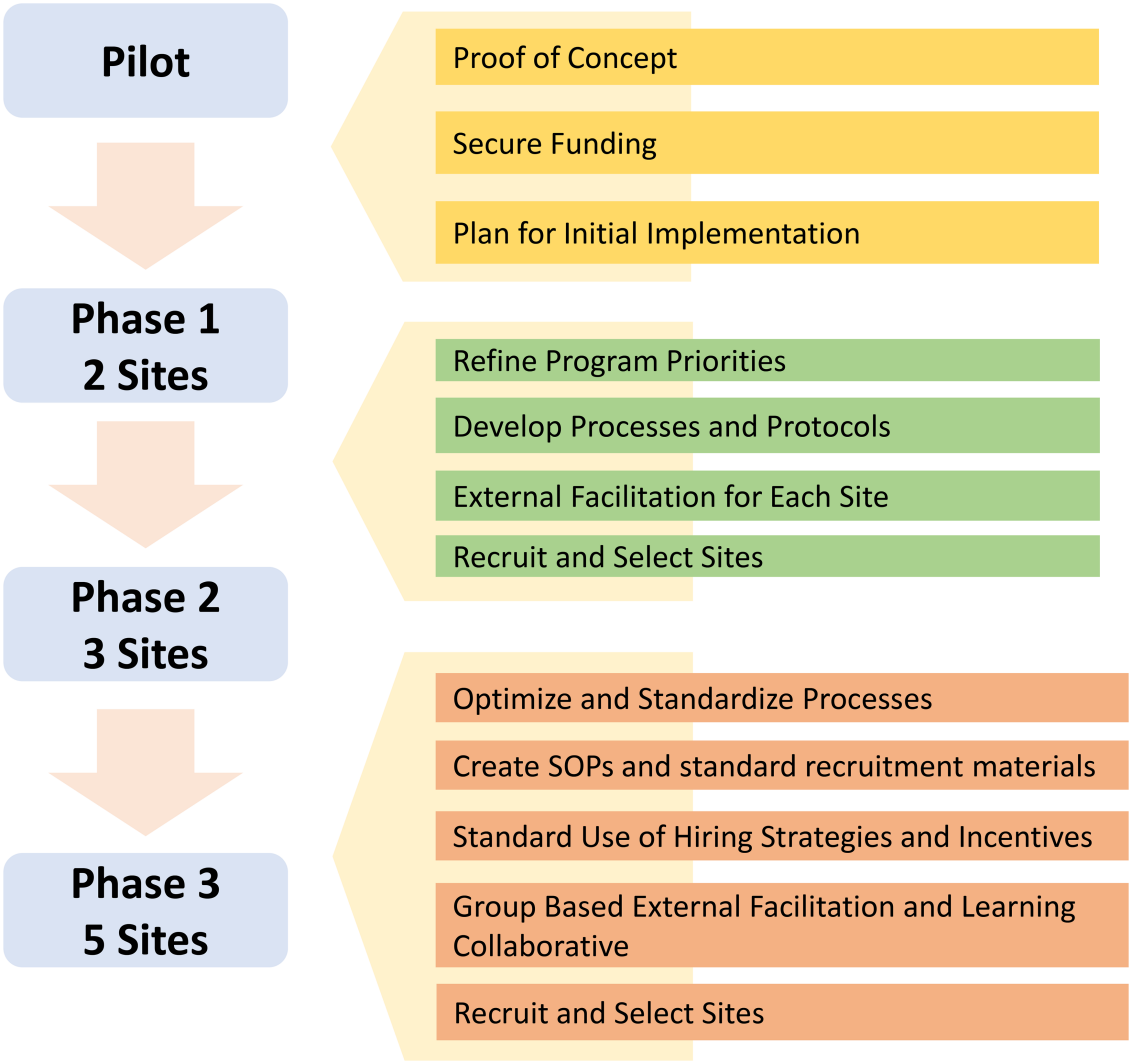
Phased MoPOC roll out timeline.

MoPOC is currently implemented at 10 VAMCs (MoPOC sites). MoPOC was implemented at these sites in a phased approach across 3 years. Figure 2 displays a timeline of MoPOC roll-out. Phase 1 consisted of 2 sites, Phase 2 consisted of 3 sites, and Phase 3 consisted of 5 sites. Centralized support is provided to all MoPOC sites by a Program Office team consisting of three staff, two clinicians who developed and piloted the program and a one administrative support person. The Office of Rural Health requires evaluation of funded programs like MoPOC. The MoPOC evaluation team is comprised of two Anthropologists, two health services researchers, a statistician, and a programmer. The evaluation team is not involved in programmatic oversight or decision making.

### Study design

We utilized the Reach, Effectiveness, Adoption, Implementation, Maintenance (RE-AIM) (11) planning and evaluation framework to assess both implementation and program outcomes. This study was conducted as part of a larger mixed methods evaluation of MoPOC implementation and program effectiveness and impact. This evaluation is a quality improvement project and not subject to institutional review board oversight. In the current study, we conducted an iterative qualitative evaluation of barriers and challenges to MoPOC implementation and adoption, as well as the strategies developed to address those challenges. Data was collected and analyzed iteratively among each of three phases. Data collection is described in detail below. Findings in each phase of data collection were summarized and shared with the Program Office Team in twice monthly meetings.

### Data collection and sources

We collected data through qualitative interviews and participant observation at meetings. We collected data across sites in phases 1, 2, and 3 of implementation between August 2020 and February 2023.

We developed semi-structured interview guides to collect detailed, salient descriptions of processes, facilitators, challenges, and adaptations during the pre-implementation and implementation periods. Interview guides included questions related to RE-AIM domains and were adaptable based on each site's stage of implementation (Supplementary Material 1). Interview guides included probing questions to help clarify participants’ responses. Table 1 provides examples of key questions and sample probes.

**Table 1:** Example interview questions.

Questions	Probes
Tell me about MoPOC implementation at your site.	*Give me an example of ____________*. *Walk me through _________*. *What happens after ________?* *What is positive about _________?* *What is challenging about _________?* *What has helped with/been helpful about ____?*
Have you/your site had enough flexibility to implement MoPOC in a way that works best?	
Tell me about your experience referring patients to MoPOC.	
Tell me how MoPOC has affected the care provided to patients	
How is the program working so far?	
Tell me about the MoPOC-related training you have received.	

We conducted semi-structured interviews with key stakeholders CPOs, PSAs, site managers, and clinicians and staff at VAMCs or CBOCs who work alongside MoPOC staff or make referrals to the MoPOC program. Interviews were conducted over video conference or in person on site visits by experienced interviewers (JY, LM, CL). Interviews conducted via video conference were audio recorded and transcribed verbatim. Detailed notes were taken during in-person interviews. Interviews were conducted continuously throughout pre-implementation and implementation phases.

We also collected data through participant observation (12, 13) at regularly scheduled meetings or check-ins between the Program Office team, clinicians, PSAs, and site managers to understand experiences implementing MoPOC and to learn about barriers, facilitators, and strategies to enhance implementation in real time. We took detailed observational field notes throughout these meetings and check-ins and asked clarifying questions to elucidate key issues that affected implementation.

### Data analysis

We used a team-based approach (JY, LM, CL) to rapid inductive/deductive content analysis (14–18). We categorized and summarized each interview in a matrix based on a-priori domains based on RE-AIM domains and project aims (such as barriers and facilitators to adoption, barriers and facilitators to implementation, implementation progress, adaptations during implementation, and perceived sustainability). Emergent domains derived directly from the data were added throughout this process. To build consensus, we summarized three interviews into domains and resolved differences in summaries through team discussion. After consensus was reached, we summarized each interview, focusing on the main points and using illustrative quotes. We entered summaries in a matrix with a row for each interview participant and column for each category to facilitate identification of patterns across interviews with attention to barriers, facilitators, and factors affecting sustainability. We discussed emergent themes at weekly analysis meetings and revisited transcripts to verify findings.

## Results

We conducted 50 interviews and 300 virtual observations at six sites. Table 2 summarizes interview participants and observations across sites.

**Table 2:** Summary of interview participants and observational data.

Type	*N*	Roles	Sites
Pre-implementation			
Interview	19	Rehabilitation Care Services Doctors, CPOs, Prosthetic Representative Chiefs, Prosthetics Clinical Managers, CPOs, Orthotist, Site Managers, Podiatrist, Health Technicians	Phase 1, Phase 2, Phase 3
Post-implementation			
Interview	31	MoPOC Program Specialist, Site Managers, CPOs, MoPOC PSAs, CBOC providers and staff, MoPOC Program Office team, Chief of Prosthetics, Amputation Rehabilitation Coordinator, physical therapy	Phase 1, Phase 2
Virtual meeting observation	300	CPO, PSAs, Site Managers	Phase 1, Phase 2

We identified four key themes related to successful program adoption and implementation, each with associated challenges and improvement strategies or adaptations: (1) “Finding the right sites for MoPOC” through intentional recruitment and site selection; (2) Identifying the “sweet spot”: Balancing program capacity, sustainability, and CPO satisfaction; (3) Shifting from testing to standardizing; and (4) “Being strategic with hiring” to improve program adoption. Table 3 briefly describes each theme and the associated strategies. Table 4 provides illustrative quotations referenced in each theme description.

**Table 3:** Theme descriptions and associated strategies.

Theme name	Description	Strategies
*Theme 1: “Finding the right sites for MoPOC” through intentional recruitment and site selection*	Learning how to choose sites best suited for MoPOC services was challenging. The first phase of sites was selected based on convenience, the second was selected based on site leadership buy-in, and the third was based on rurality and locations of CBOCs that would be serviced	• Developing scoring criteria based on existing services at a site, location and population density of rural CBOCs, and leadership commitment to supporting MoPOC • Developing and disseminating informational materials about what is needed for a MoPOC site • Providing informational sessions with question-and-answer sessions during application process ○ Improving site selection criteria Rurality ○ Location of CBOCs ○ Ability to adhere to MoPOC program model
*Theme 2: Identifying the “sweet spot”: Balancing program capacity, sustainability, and CPO satisfaction*	It is difficult to serve as many Veterans as possible and optimize the services delivered while ensuring program sustainability and protecting the CPO from burn-out.	• Identifying program guidelines around maximum drive times • Providing guidance around number of encounters per month • Guidance around scheduling to reduce administrative burden • Providing guidance around scope and complexity of services
*Theme 3: Shifting from “testing to standardizing”*	The first 2 years of roll-out were spent refining the program model, developing best practices, and writing Standard Operating Procedures. In the third year, new guidance was provided to sites in order to standardize how MoPOC is delivered across sites.	• Setting expectation of 50 clinical encounters per month with 1-hour appointment slots • Asking that clinician and staff schedules align across sites • Providing guidance around CPO/PSA working relationship • Suggesting number of service days at each CBOC • Stipulating that MoPOC sites must expand overall capacity to provide services, not simply realign existing staff to cover new locations.
*Theme 4: “Being strategic with hiring” to improve program adoption*	Hiring qualified clinicians in extremely rural areas can be challenging. Slow turnaround with human resources and a limited pool of qualified candidates resulted in long hiring timelines and delayed start of services at sites in the first two phases.	• Providing centralized training and guidance on hiring processes • Refining position descriptions • Providing one-on-one and group-level guidance throughout the hiring process • Using relocation and retention incentives • Using centralized CPO advertising and recruitment

**Table 4:** Illustrative quotations.

Quotation number	Quotation
Q1	*When selecting new sites, there is a need to balance the need to serve rural Veterans with good drive times. [Site name] and [Site name] are not the best sites for this. -Program Office Team Member*
Q2	*We also look at the environment around the site and whether the environment is well suited for MoPOC…do they have CBOCs that fall within a 100 mile radius, and is the population density high enough that you can keep one clinician busy by going to one or two CBOCs? The tricky piece is that if you are targeting rural centers you might have a location that has the perfect anchor site and the CBOCs are the perfect distance, but the population density is so low that you can't keep one person busy. It took awhile for us to understand both spheres- what the anchor site looks like and what environment is right for providing mobile care to the CBOCs. -Program Office Team Member*
Q3	*Our initial fear was that we would select a site that was poorly run so we talked to people who had been in the VA a long time and asked them to tell us which were well-run VAs that were located in rural areas. So our criteria as we expanded in our second year were only based on a loose understanding of the environment and a loose understanding of what it meant to have a well-run facility. We have developed an understanding over time to ask that question and determine how to tell if a site is well run. -Program Office Team Member*
Q4	*We’ve learned that lesson…leadership can say “sure we support MoPOC, it's a great idea,” but without a signed agreement about what MoPOC takes to get up and running…we don't have any way to hold them accountable to that. -Program Office Team Member*
Q5	*Now [we] have developed a series of memos for a range of leadership including from CBOCs, Fleet, Logistics and HR] that are statements that are written out that say, we need you to agree to participate. By requiring sites to have those memos signed we now have built a coalition of leadership support that can help the site be successful. We always knew leadership support was important but we didn't fully understand how to garner that support or understand the level of detail that needed to be in those memos to remind people that they had agreed to provide this support. -Program Office Team Member*
Q6	*We did info sessions last year for the first time and we did them again for this cycle. I think they have been really useful. They tend to be very well attended. I think collectively we have had over 100 attendees to this cycle's info sessions. I think it gives applicants a good idea of what is involved in the program and I think probably selects sites out…helps sites understand when they might not be a good fit. -Program Office Team Member*
Q7	*Since we have a really good understanding of what makes a good site from an environment perspective, we’re now building a spreadsheet with distances to CBOCs so we can understand how many environments there are that would work for our model, and we’re reaching out to those sites. There is a balance of sites requesting information and us requesting sites to meet with us to learn about the program. -Program Office Team Member*
Q8	*I know I’m just hurting myself but I try to squeeze them in when I’m here [at CBOC]. If I don't they will have to wait a week maybe more…and sometimes they are right in front of me asking for help and there is no way I can say no…so I just do it and then deal with the fall out later…one more person to chart, order, it adds up and just puts me more behind…but my time is valuable when I’m out here so I see as many people as I can. -Phase 2 CPO*
Q9	*Everything takes longer at the CBOCs…doing the work, seeing the Veteran, ordering, scheduling…if I had to guess I’d say it takes me 30% more time to do the same work…I don't have the same tools, the same space, the same staff. -Phase 1 CPO*
Q10	*Any problems get amplified on the burdensome side because you are by yourself, in-office clinicians have more buffer time for administrative or lab tasks. If you run over on time with a patient, there is no back up, it is just you and you have to plan for the rest of your travel and plan ahead, you can't take for granted you have a full lab and people as resources to fall back on. -Phase 1 CPO*
Q11	*I’m seeing a lot of complicated patients, very complicated…I wish I had more time but it's just all spent on patient care…what suffers is my notes and things like ordering…[I am] always struggling to get those done. -Phase 1 CPO*
Q12	*It's a balance between wanting to keep the complexity of care high but don't want to burn out the CPO. People underestimate the driving and other work that goes into being a mobile clinician…spending time building relationships, getting to know personalities…not just clinical work. -Phase 2 Site Manager*
Q13	*[When we first started MOPOC] we wanted to focus on complex devices. It turns out that was a mistake. [It] wasn't sustainable. [There was a] “mismatch” between number of encounters and complexity, just couldn't do it…We needed to adjust to a more balanced approach. -Program Office Team Member*
Q14	*The system needs to help us with finding the balance, someone needs to be triaging to help us make the most of a finite resource, but it can't be me…so who will do it…and who will say no to the Veterans that we don't have time or space to see? -Phase 1 CPO*
Q15	*We need to help you make this workable…make sure that you are able to get your work done timely, pull back on the number of encounters so that you can get all the care done in your day…so you don't get burned out. -Program Office Team Member*
Q16	*We’d love to have someone out at [two CBOCs] but we’d have to weigh the cost and benefit of that. It is a 3 h drive from [anchor] to [CBOC], so that could make it a 12-hour day for someone…it doesn't really make sense in terms of the payoff and would be a brutal drive for a clinician. -Phase 2 Site manager*
Q17	*If you are spread so thin it doesn't really help anyone…you aren't in any one place enough to really offer care fast enough to meet needs…[patients] end up waiting and you feel bad that you can't get them the things they need when they need them. -Phase 1 CPO*
Q18	*It's frustrating to have your care limited by weather and have to cancel on people…it's a reality when you are going across mountains but it ends up in more delayed care…the next time you are out there you are going to be working double time to catch up. -Phase 1 CPO*
Q19	*We’ve been able to identify, with your help [evaluation team] what MoPOC needs to be successful…we’ve been able to more confidently say “this is how you have to do it” and feel like we now have the data and experience to back it up. -Program Office Team Member*
Q20	*This isn't just a handout…here's a bunch of money and a van, now do what you want…so we are trying to balance our oversight and expectations with leaving some space for sites to make their own decisions on the ground that work for them. -Program Office Team Member*
Q21	*It feels very organized for the second year of people. First year, it feels like we’re still in the middle of building the plane while it's in the air.” -Phase 2 Site Manager*
Q22	*This will be a new position for both [the PSA] and the [clinician] and in the beginning they will basically be working together to create an office space/workflow that works for their site all by trial and error. -Phase 1 PSA*
Q23	*We made the decision to have them [PSA] at [a different VAMC than the clinician]…we knew it might not be optimal…it has turned out to be a really bad decision…affected communication, how much they can help the clinician…wish had known how much it would affect our program. -Phase 2 Site Manager*
Q24	*Well, I don't have any support here. I mean [medical support assistants] are great, but they can't do work for me. They can't, you know, sometimes I ask him to bring stuff down to the mail room when it needs to go down on a Friday. But, you know, packing a box or organizing something or, you know, that's not, that's not their job. -Phase 2 CPO*
Q25	*I’m glad that what we are doing is making it better and easier for the next round of sites…appreciate that we are listened to and that [program office team] really listens to our experiences. -Phase 2 Site Manager*
Q26	*We don't have a lot of practitioners out here because we are rural and VA…makes it harder because you have to have certain credentials and experience…way beyond that in the private sector…so hard to find anyone who wants to live here and we can hire…that's not MoPOC, that's just VA. -Phase 2 Site Manager*
Q27	*I don't have any experience with this stuff [hiring]…thank goodness [the program office team] is holding our hands and showing us how to do it…just saved me 2 days of work just by giving me the right forms to use. -Phase 2 Site Manager*
Q28	*My biggest need is the hiring…need someone to grease the wheels to move the hiring along…somebody who can twist arms at HR…They see my name and that isn't going to make them say “hey we need to listen to this guy.” -Phase 2 Site Manager*

### Theme 1: “finding the right sites for MoPOC” through intentional recruitment and site selection

The Program Office team described the process of refining site selection criteria. In the first phase of implementation, they discussed selecting sites that they were familiar with so they could learn how to implement and manage MoPOC sites (“*If you loop back to our very first sites…those sites were local sites that we managed because we needed to learn how to manage a site*”). At those sites, they described that it was challenging to serve a rural population while also ensuring that clinician drive times were reasonable (Q1). The CBOCs with the highest rural populations were prohibitively far from the anchor VAMC or did not have enough patients who needed O&P services to keep a CPO busy. Based on these challenges, they learned that site criteria should address the need to balance rurality, drive times, and density of rural patients who need O&P services (Q2).

However, they described that rurality and CBOC location were not the only considerations in selecting MoPOC sites. When selecting sites for Phase 2, they described their apprehension around selecting sites that might fail to implement MoPOC successfully. They felt that it was important to select “well run” sites to enhance program success, and described that they did not have a clear idea what it meant for a site to be “well-run” at that time (Q3). Based on their experience selecting sites for Phase 2, they learned that hands-on leadership support is critical to successful adoption and implementation. They described that leaders may state their support during the application process, but then fail to provide support when challenges arise (Q4).

One of the primary strategies developed to improve site selection centers around galvanizing and ensuring leadership support at MoPOC sites. The Program Office team discussed the importance of executive-level leadership support, as well as the support of leadership from participating CBOCs, the vehicle fleet, contracting, and logistics to ensure that CPOs have sites at which to provide care, a vehicle to drive, and the tools they need to fabricate patient devices. To ensure this support, the Program Office team developed Memoranda of Understanding (MOU) that clearly describe the roles and responsibilities of involved parties, each requiring signatures from critical stakeholders (Q5). Though not legally binding, MOUs carry significant weight within VA and essentially act as a guarantee of commitment.

The other primary strategy centers around raising program awareness and recruiting sites. The Program Office team regularly attends and presents their work at large recurring virtual meetings of VA Prosthetic leadership and field staff. For several years they have also shared their work at the annual VA Breakout, which takes place adjacent to the nation's largest research-oriented O&P conferences. By presenting at these meetings, they raise awareness, socialize the program model, and continually highlight the characteristics that lead to success for prospective sites. As part of their annual application process, the Program Office hosts two information sessions which provide an opportunity to introduce the MoPOC care model, discuss what is expected of MoPOC sites, and explain what makes a competitive application for selection as a MoPOC site (Q6). Finally, Program Office team members described strategies to proactively identify sites that might support the MoPOC care model and outreach directly to Prosthetics leaders at those facilities (Q7).

### Theme 2: identifying the “sweet spot”: balancing program capacity, sustainability, and CPO satisfaction

All participant groups described implementation challenges related to scaling MoPOC services and challenges around balancing program reach with sustainability and clinician satisfaction. Descriptions of these tensions focused around two primary domains: (1) balancing program capacity with clinician satisfaction, and (2) seeing the “right patients in the right places.”

#### Subtheme 1: balancing program capacity with clinician satisfaction

Participants described several reasons that providing MoPOC care was more demanding for the clinicians than traditional O&P care out of a VAMC. While providing care at CBOCs, clinicians described several demands on their time in addition to scheduled patient care. These included educating CBOC providers about available O&P services, “building up relationships” with CBOC providers by accommodating requests for unscheduled same-day care, informal consulting with CBOC providers, and warm handoffs of patients with immediate needs from other CBOC providers. They also described that accommodating walk-in patients impacted their schedule and made it difficult to keep up with their administrative workload (e.g., writing notes about patient visits, ordering supplies, following up with patients) (Q8). In addition to experiencing extra demands on their time while providing care at CBOCs, clinicians indicated that providing mobile O&P care is inherently more time consuming because existing processes within VA were not created for mobile clinicians. Mobile clinicians do not have the same space, tools, or support staff while at CBOCs (Q9). A mobile clinician will likely need to walk out to their vehicle to make modifications to a device, while a clinician at a VAMC would only need to walk down the hallway to a well-appointed lab space. Finally, CPOs described working alone with no back-up. This was challenging due to a lack of buffer time between appointments, no team members to help carry the workload if a patient encounter runs long, and the need to plan for travel. They described that scheduling issues could amplify these problems (Q10).

Despite the challenges associated with providing mobile care, clinicians described wanting to help as many patients as possible, and especially patients with complex needs (Q11). Site managers described the tension between wanting to maximize clinical capacity and provide MoPOC services to the patients who need them most while also ensuring that clinician schedules are sustainable. They worried that challenging schedules increased the risk of burnout for CPOs (Q12). The Program Office team was aware of this tension. They described that among Phase 1 sites, they set an expectation that clinicians see a pre-determined number of clinically complex patients each month. They felt that seeing a high volume of patients would satisfy the funder, but learned that focusing too much on the number of patient encounters led to clinician burnout (Q13). Clinicians also described concerns around burnout, and that “the system” needs a way to help CPOs find balance (Q14).

Strategies to mitigate challenges around optimizing program capacity and scope of care included adapting, refining, and improving mobile care workflows and modifying expectations to improve sustainability of the clinician role. Participants summarized changes in program expectations including a reduction in the target number of monthly encounters, ensuring that clinicians have 1 day each week dedicated to administrative tasks, deepening the partnership between clinicians and their PSAs to ease administrative load on clinicians, and establishing learning collaboratives where clinicians and PSAs can share experiences and problem solve. In addition, the Program Office team began meeting with clinicians regularly to learn about their experiences, offer support and guidance, and serve as advocates. The Program Office team described that their role was to help make the clinician role “workable” (Q15).

#### Subtheme 2: seeing the right patients in the right places

Participants described that selecting locations for MoPOC care and patients for MoPOC care needs to be intentional. Because MoPOC is funded by the VA Office of Rural Health, the program is focused on meeting the needs of rural Veterans. In many cases, this means traveling to the CBOCs that are furthest from VAMCs in locations where few other services are available. In other cases, this means providing care to Veterans in their homes. Participants stated that long drive times and travel to several locations can impact sustainability and reach. Long drives are taxing on clinicians and reduce the time that they can dedicate to patient care (Q16). They described that trying to provide care at too many locations results in the clinician being “spread thin” and that it impacts the clinician's ability to provide timely care with adequate ensure continuity (Q17). Further, because MoPOC services are delivered by a single clinician at each site with no back-up, weather, illness, and vacations can disproportionately disrupt care continuity (Q18).

Strategies to help identify appropriate locations for MoPOC care centered around defining maximum drive times for clinicians, identifying CBOCs that serve a high volume of rural patients, providing guidance around the frequency of visits to each CBOC, and monitoring program rurality metrics. Based on feedback from the clinicians, the Program Office team now suggests that CBOCs be located within a 1.5-hour drive of the anchor VAMC. They also ask that clinicians visit each CBOC at least once per week to ensure continuity of care. The Program Office team also helps sites build demand for MoPOC services at rural locations that have less full schedules. They provide flyers, help with networking, and suggest providing education on O&P services to CBOC clinicians to build the number of referrals. Finally, the Program Office team works with Site Managers, clinicians, and PSAs to monitor and review data on encounters each month to see how sites are meeting goals of serving rural Veterans, timeliness of care, and best use of resources. In cases where the data and clinician feedback suggest that a particular CBOC is not appropriate for MoPOC, the Program Office team and site will consider changes to schedules and service locations.

### Theme 3: shifting from “testing to standardizing”

The Program Office team and Site Managers described shifting from a testing and “building as we go” approach to greater standardization across sites. Program refinement and adaptation at a central level occurred over time, through testing, reflection, and iteration. The Program Office team described that when MoPOC was implemented at Phase 1 sites, they were still defining the program model. They had not yet developed tools or infrastructure to support implementation. After testing processes and the core components of MoPOC, they developed new processes for onboarding sites and worked to bring existing sites into compliance. They described the general need for standardization so that “we can recognize MoPOC across VA”; we can say “yes, this is MoPOC” (Q19). To increase standardization, they have developed stipulations that come along with MoPOC funding (Q20). A Phase 2 Site Manager described the process of standardization from the MoPOC site perspective, stating that roll-out felt very organized for Phase 3 sites, whereas it was still in a testing phase for Phase 2 sites (Q21). Though participants described several ways that MoPOC was standardized over time, we focus on standardization of the Program Support Assistant (PSA) role to improve delivery of MoPOC services.

#### PSA role

The MoPOC PSA provides administrative support for clinicians. Site Managers and clinicians describe PSAs as essential to the efficiency and success of MOPOC. Despite the importance of the PSA role, all participants discussed challenges around defining the PSA role. While medical support assistants (MSAs) are common in O&P services, PSAs are not. Participants described that the PSA role involves more responsibility and problem solving around scheduling, triaging patients, ordering and tracking, care communication, communication with vendors and Veterans, administrative management, and setting up new administrative work-flows and systems. PSAs described challenges with the PSA role, including that both clinicians and PSAs will be beginning a new role and will need to work together closely to create work flows for their site (Q22).

In phases 1 and 2, sites independently made decisions about PSA location, schedule, and whether to have dedicated PSAs for each clinician. The Program Office team did not set expectations around PSA work location or schedule. This led to differences in implementation among sites and varying success in the clinician/PSA relationship (Q23).

Strategies to standardize the PSA role included defining the role, detailing how the clinician and PSA should work together, and developing a PSA learning collaborative. The Program Office team worked with the Phase 1 PSA to draft a detailed description and performance plan for the PSA role and to develop expectations for how the clinician and PSA should work together. Based on feedback from Phase 1 and 2 sites, the Program Office team also decided that each clinician should have a dedicated PSA, rather than 1 PSA to 2 CPOs as originally conceived, and that the clinician and PSA should work from the same anchor site to allow for daily check-ins and in-person communication. One clinician described challenges associated with not having a dedicated PSA, including difficulty scheduling and dealing with orders and supplies (Q24).

Participants also described additional strategies used to move from the testing to standardization phase. These included documenting best practices and developing standards of practice, involving sites in discussions around testing and standardizing, deferring to site-level experience and expertise, creating learning collaboratives so that new sites can learn from the experiences of sites that have already implemented, and using evaluation data and feedback to inform program improvement (Q25).

### Theme 4: “being strategic with hiring” to improve program adoption

The Program Office team and Site Managers described hiring as the biggest barrier to adoption and implementation of MoPOC. Challenges related to hiring included difficulty finding qualified clinicians in rural areas, slow VA hiring timelines, and the fact that VA is often not competitive with the private sector in terms of salary and benefits. Further, VA has strict credentialing guidelines that limit the pool of candidates (Q26). In addition, some Site Managers described their lack of familiarity with hiring processes as a barrier to hiring. One talked about the need for support from the Program Office team during the hiring process (Q27).

Other participants discussed challenges with hiring that stem from MoPOC being a grant funded program. They described that because funds need to be spent within certain windows of time, failure to successfully onboard personnel within specified timelines can be detrimental; if the program is not implemented the site will lose the funding. One Site Manager described the challenge of working with HR when hiring timelines are tight (Q28). The Program Office team described reaching out directly to site-level human resources staff to help expedite hiring on behalf of local Site Managers, “*we can be the ones to put pressure on the system […] we can be the bad guys.*”

Strategies to alleviate hiring challenges at new MoPOC sites focused on working with human resources to improve hiring timelines and strategies to recruit qualified clinicians. To help expedite the recruitment and hiring process, the Program Office team now provides support to Site Managers through coaching sessions, provides email templates and communication strategies, and also communicates directly with human resources supervisors or leadership to highlight the importance of hiring timelines to the grant funded position. A Phase 2 Site Manager described the utility of intervention from the Program Office team, “*[a Program Office team member] wrote an email underscoring the urgency of hiring … [and I saw] instant progress.*”

Strategies to recruit clinicians include the use of incentives and centralized advertising about available MoPOC positions. Participants described that it can be difficult to hire candidates in rural areas due to both lack of candidates that meet VA requirements and lack of reasons for people to move to the area. After failing to hire a candidate the first time the position was posted, a Phase 2 site described re-posting the position with incentives like a hiring bonus and relocation bonus. This resulted in hiring a candidate who met both VA criteria and program needs. The Program Office team suggested a complementary strategy of paying the hiring bonus in installments to improve retention of clinicians in the position. Due to the success of these strategies, Phase 3 sites were advised to add incentives to the CPO position after a single unsuccessful round of posting, and consequently, those positions have been filled more rapidly than in earlier phases. The Program Office team also now advertises open MoPOC CPO positions in a national news outlet intended for O&P clinicians and administrators.

## Discussion

The goal of this study was to understand the challenges and mitigation strategies developed in the phased rollout of MoPOC across VA. Implementation and scale-up of mobile O&P care is not well described, and the experiences of the MoPOC Program Office team, clinicians, and key stakeholders can help inform strategies for implementation and dissemination of new mobile O&P programs or other mobile specialty care in both VA and non-VA settings. Our findings suggest that there are several important considerations in developing and implementing mobile O&P services.

A large body of work suggests that local context influences implementation of new programs (19–21). The MoPOC Program Office Team developed strategies around site selection to enhance MoPOC adoption and implementation. These strategies focused on selecting sites with strong O&P services and demonstrated leadership support. The importance of leadership support in implementing evidence-based practices is well described (22–24). Our study adds nuance to understanding what types of leadership support are needed for successful implementation of mobile O&P care. While executive leadership support was needed, support from leadership at participating clinics, fleet management for vehicle procurement and support, and human resources were also essential. The other consideration around site selection related to site-level need for MoPOC services. The Program Office Team and Site Managers discussed how site selection could ensure that MoPOC has the greatest impact on access to care for rural Veterans. They determined that the anchor site should serve clinics with a large rural population within a reasonable driving distance from the anchor site. This intentional site selection ensured that the substantial resources needed to implement a MoPOC will be allocated to sites that are likely to successfully implement. Site selection may be similarly important in non-VA contexts. Implementing mobile O&P care will have greatest success in settings with documented leadership buy-in and high patient need for O&P care.

Findings around balancing program capacity and clinician satisfaction are critical when thinking about scaling MoPOC to each individual setting. Clinician burnout is a recognized problem (25, 26), and previous work suggests that clinicians in rural areas experience increased levels of burnout relative to those in urban centers (27). Strategies developed to appropriately scale MoPOC and alleviate risk of clinician burnout included developing guidelines around how often a clinician should visit each care site, how many care sites are appropriate, how far those care sites should be from the anchor VAMC, how many patient encounters are feasible, and how much time should be devoted to each encounter. Expectations around number of encounters for CPOs may be lower than those for their counterparts providing O&P care in hospital settings to allow for extra time needed to work remotely from rural locations. These are important considerations when determining necessary resource allocation to mobile O&P programs in both VA and non-VA settings. While decisions around program sustainment often hinge on economic analyses (28–30), mobile O&P care is likely less efficient and the costs of mobile O&P may be greater than costs of traditional, hospital-based O&P care.

The shift from testing to standardizing MoPOC reflects considerations in scale-up that have been previously described (31–33). First, in order to scale-up a program, there needs to be a defined scalable unit (32). After an initial testing phase, defining the MoPOC scalable unit required iterative feedback and reflection from Program Office Team members, Site Managers, CPO's, and PSAs. Designing a program for scale-up involves defining the key players and developing and testing protocols (31). In MoPOC, protocols were developed collaboratively across phases leading to adaptations in how MoPOC was implemented over time.

Finally, our finding that strategic hiring practices were needed to improve MoPOC adoption reflect a broader trend. Clinician shortages in rural areas are well documented (34–37). In MoPOC, these challenges were compounded by VA's strict credentialling requirements and by the need to recruit clinicians willing and able to travel to provide care. Previous work suggests that hiring delays at VA can cause delays in implementation (38). The MoPOC strategy of using incentives to attract qualified candidates improved the time that it took to fill CPO positions and begin serving rural patients in need of O&P services. While non-VA settings may have different hiring procedures and challenges, offering hiring and retention incentives may enhance hiring for O&P clinicians in other rural contexts.

Importantly, the challenges we identified related to implementing and scaling up a mobile O&P program are likely relevant to implementing other forms of specialty care in rural clinics. VA recognizes the need to offer specialty care outside of VA medical centers (39), and there is a need to understand the feasibility and impacts of offering specialty care in different locations. Challenges with access to specialty care in rural areas are well described, and programs like MoPOC that can circumvent these challenges have the potential to transform the types of care that rural patients can access close to their homes. Our findings suggest that programs should carefully consider location and demand for services, clinician drive times, and realistic expectations for clinician schedules and number of encounters.

The RE-AIM framework provided a standard reporting framework within which to identify challenges, mitigation strategies, and the impacts of those strategies across implementation phases. In many contexts, RE-AIM is used to summative assess implementation and program outcomes (40), but in an ongoing evaluation context iterative cycles of data collection, adaptation, and data collection have the potential to continuously update implementation strategies or program delivery to best suit new or changing clinical contexts. Though this study focuses explicitly on MoPOC implementation, improvement in the delivery of MoPOC care impacts patient experience, and future work should assess patient satisfaction across implementation phases. Using the RE-AIM framework to evaluate clinician, Program Office team, and stakeholder experiences implementing in successive phases of scale-up has strengthened delivery of MoPOC in several ways. First, comparing findings across phases of implementation underscores common challenges in implementing mobile specialty services in diverse VA settings and identifies strategies to address those challenges. Second, iterative use of RE-AIM allowed the MoPOC Program Office team to use evaluation data to improve program delivery in each phase of expansion, and then understand how those adaptations affected MoPOC implementation. Finally, evaluating each successive phase of implementation allowed the evaluation team to be responsive to program needs and understand which stakeholders and time points would optimize collection of salient, actionable information around MoPOC implementation.

## Limitations

This was a qualitative study and findings should be interpreted in the context in which they were collected. The experiences and perceptions of the CPOs and stakeholders shared in this work may not be representative of all stakeholders. Future work should assess the impact of MoPOC on patient satisfaction with O&P services within VA. Because this was a quality improvement study, findings are not meant to be generalizable. The challenges and implementation strategies developed to address those challenges may be relevant for implementation and scale up of other mobile specialty care services.

## Data Availability

The datasets presented in this article are not readily available because we are unable to provide full de-identified interview transcripts as this was not discussed with participants during the consent process. Requests to access the datasets should be directed to the corresponding author.
