# Supplementary material for: Implementation of a mobile prosthetic and orthotic care program in the VA; a qualitative study of implementation challenges and associated strategies for improvement
Source: Front Health Serv. 2024 Nov 1;4:1198191. doi: 10.3389/frhs.2024.1198191 (PMC11564175; doi:10.3389/frhs.2024.1198191)
Supplement: Supplementary file 1 [file Datasheet1.docx]

**MOPOC Pre-Implementation Interview**

Script:

Hello [Dr./Mr./Ms. interview participant name],

My name is [interviewer name] and I am part of the team tasked with conducting an evaluation of the MoPOC [Mobile Prosthetic and Orthotic Care] program. We are interviewing key stakeholders to understand how prosthetic and orthotic care currently works at your facility and the best way to roll out MoPOC at your site.

Participation in this interview is **voluntary, anonymous, and confidential**. You do not have to participate in this interview. If you choose to participate you can stop the interview at any time and do not have to answer any questions you don’t want to. We won’t identify you as a participant or identify your site [if applicable] in any of our reports to the Office of Rural Health.

The interview will take approximately 30-45 minutes.

Do you have any questions?

In order to make sure we capture all of the information you give us, we would like to record this call. The audio-file for the recording will be uploaded to a restricted access file on the VA intranet immediately after we complete this interview. The audio file will be saved anonymously. We may transcribe the recording, and your name will be removed from any transcripts.

Is this okay with you?

***Grounded prompts: If responses are limited or require clarification, probes may be used to illicit more detailed responses. Probes should use words or phrases presented by the participant using one of the following formats:***

1. *What do you mean by ____________?*
2. *Tell me more about ____________.*
3. *Give me an example of ____________.*
4. *Tell me about a time when ____________.*
5. *Who __________?*
6. *When __________?*
7. *Walk me through _________.*
8. *What happens after ________?*
9. *How does [x] compare with [y]?*
10. *What has gotten in the way of ____________?*
11. *What has helped with _____________?*
12. *What is positive about _________?*
13. *What is challenging about _________?*
14. *How has _______ changed?*

T**ell me about your role at (name of facility)**

If needed: Tell me what you do at VA. How you work with Veterans with O/P needs.

If needed: How are you involved with O/P care?

What types of O/P needs do Veterans have?

**Tell me about the prosthetic and orthotic services offered at this facility/in your department.**

- - Are there services that are not offered?
  - Have there been changes to the services offered in VA?
  - Where are these services offered?
  - How are these services offered (in-person, telehealth, phone, other)?

**Please walk me through the current process of prosthetic and/or orthotic care at your site.**

*(RE-AIM; assessing Adoption and Implementation needs)*

- What is your role in the process?
- Who are other important players in this process?
- What works well about the current process?
- What does not work well about the current process?
- *If needed:* Tell me about the process for getting Veterans prosthetic/orthotic care in the community.
- *If needed:* Tell me about the process for getting Veterans prosthetic/orthotic care in VA.

**How successful is the current process of prosthetic and/or orthotic care?**

*(RE-AIM; assessing Adoption and Implementation needs)*

- What contributes to this success/lack of success?
- What factors affect your site’s ability to provide prosthetic/orthotic services to Veterans?
- What, if anything, should be changed?

**How easy is it for Veterans to get their O/P needs met?**

*(RE-AIM; assessing perceived potential Reach and Effectiveness)*

- - In VA?
  - In the community?
- What challenges, if any, do Veterans have in accessing the prosthetic and/or orthotic care they need?
  - In VA?
  - In the community?
- *If needed*: Tell me about the timeliness of prosthetic and orthotic care that Veterans receive.
- *If needed*: Tell me about the quality of prosthetic and orthotic care that Veterans receive.
- *If needed*: Tell me about care continuity and coordination for Veterans with prosthetic/orthotic needs.

**What, if anything, have you heard about MoPOC?**

*(RE-AIM; assessing perceived Effectiveness)*

*MoPOC, which stands for Mobile Prosthetic and Orthotic Care, is a new VA effort to offer state of the art, accessible prosthetic and orthotic (P&O) services to rural-based Veterans and those who experience barriers traveling to VA facilities. MoPOC will bring VA P&O specialty care to selected VA CBOCs and to Veterans’ homes via a mobile unit. Mobile units will be staffed by VA Certified Prosthetists/Orthotists and equipped with a suite of tools and technologies provide onsite care including the fabrication, modification and fitting of custom prosthetic and orthotic devices.*

**What do you think about this program?**

*(RE-AIM; assessing perceived Effectiveness, needs for Implementation)*

- What ways, if any, could it be beneficial?
- What ways, if any, could the new models be challenging?
- What concerns, if any, do you have about the new models?
- Do you think you would refer Veterans to the MoPOC program? Why or why not?
  - Which Veterans would be best served by MoPOC? (eg who are the ‘right’ patients?)
- Do you think the Veterans you work with would be interested in MoPOC? Why or why not?
- What is the best way to inform providers/people at your site about the MoPOC program?

**Have you had any contact with the program or clinicians?**

**What impact, if any, do you think MoPOC would have on the way prosthetic/orthotic care works at your site?**

*(RE-AIM; assessing perceived Adoption and Implementation needs)*

- How would MoPOC work best at your site?
- How do you envision interacting/interfacing with MoPOC?
- Are there any competing priorities that may affect the roll out of MoPOC at your site?

**What, if anything, could get in the way of successfully implementing MoPOC at your site?**

*(RE-AIM; assessing perceived Adoption and Implementation Challenges)*

- Do you have any suggestions for addressing or overcoming these barriers?

**What will be key to successfully implementing MoPOC at your site?**

*(RE-AIM; Implementation)*

**How ready is your site for MoPOC implementation?**

*(RE-AIM; assessing perceived Effectiveness)*

- Where will MoPOC services be located/delivered?
  - Will MoPOC prosthetists/orthotists be housed at any CBOCs or other VA locations?
- What steps need to occur?
- Who is involved or needs to be involved?
- Do you/does your site have the resources needed to implement and support MoPOC?
  - If yes, please describe.
  - If no, what resources would you like to receive?

**What would MoPOC program success look like?**

*(RE-AIM; Effectiveness; Implementation; Maintenance)*

- For providers?
- For patients?
- How will the decision to sustain or not sustain MoPOC be made?
- In thinking about sustaining MoPOC at your site, what factors will be considered?
- What data would be needed to make a decision on permanently adopting the program?
- Who will make the decision about sustaining MoPOC once the pilot is completed?

**Is there anything else you would like us to know about VA O/P care or the MoPOC program?**

**Is there anyone else we should talk to in order to understand O/P care or to get feedback on the MoPOC program?**

Thank you very much for your time. We really appreciate you sharing this information with us.

**MOPOC Post-Implementation Interview**

Hello [Dr./Mr./Ms. interview participant name],

My name is [interviewer name] and I am part of the team tasked with conducting an evaluation of the MoPOC [spell out] program. We are interviewing key stakeholders to understand program roll out, people’s experience with MoPOC, and its impact on clinicians, staff, and Veterans.

Participation in this interview is **voluntary, anonymous, and confidential**. You do not have to participate in this interview. If you choose to participate you can stop the interview at any time and do not have to answer any questions you don’t want to. We won’t identify you as a participant or identify your site [if applicable] in any of our reports to the Office of Rural Health.

The interview will take approximately 30-45 minutes.

Do you have any questions?

Would you like to participate in this interview?

- If no, thank person for their time and end call.
- If yes, continue:

In order to make sure we capture all of the information you give us, we would like to record this call. The audio-file for the recording will be uploaded to a restricted access file on the VA intranet immediately after we complete this interview. The audio file will be saved anonymously. We may transcribe the recording, and your name will be removed from any transcripts. Is this okay with you?

***Grounded prompts: If responses are limited or require clarification, probes may be used to illicit more detailed responses. Probes should use words or phrases presented by the participant using one of the following formats:***

*What do you mean by ____________?*

*Tell me more about ____________.*

*Give me an example of ____________.*

*Tell me about a time when ____________.*

*Who __________?*

*When __________?*

*Walk me through _________.*

*What happens after ________?*

*How does [x] compare with [y]?*

*What has gotten in the way of ____________?*

*What has helped with/been helpful about _____________?*

*What is positive about _________?*

*What is challenging about _________?*

1. *How has _______ changed?*

T**ell me about your role at (name of facility).**

**Tell me about the MoPOC (Mobile Prosthetics and Orthotics) Program.**

- How did you hear about MoPOC?

**Tell me about MoPOC implementation at your site.**

*(RE-AIM; Implementation)*

- What are current MoPOC activities at your site?
- What has not been able to be/still needs to be implemented?
- What strategies, if any, have you or your team used to get MoPOC up and running/fully implemented?
- How has the MoPOC roll-out gone so far?
- What are the next steps going forward?
- What lessons have been learned?
- Who are the key people involved in MoPOC activities, efforts, and discussions?
  - What have been their primary concerns, hopes, or suggestions?

**Have you/your site had enough flexibility to implement MoPOC in a way that works best?**

*(RE-AIM; Implementation)*

- What aspects of the program were changed/adjusted?
- What aspects of the program would you like to change, but can’t?

**Tell me about your experience with MoPOC.**

*(RE-AIM; Effectiveness, Implementation)*

- What interaction or communication, if any, have you had with the MoPOC program or clinicians?
- To your knowledge, have any of your patients received MoPOC care?
- What feedback, if any, have you received about patients’ experiences with MoPOC?
- How, if at all, has your practice or work changed or been affected by MoPOC?
- What has gone well or has been helpful?
- What has not gone well or has not been helpful?
- How beneficial is MoPOC:
  - to Veterans? To your site? To VA?
- If applicable: What benefit, if any, is there in having a prosthetist/orthotist at your CBOC?
- If applicable: What downside, if any, is there in having a prosthetist/orthotist at your CBOC?

**Tell me about your experience referring patients to MoPOC.**

- Walk me through referring a patient.
- What types of patients do you/would you refer to MoPOC?
- Are there patients you do not/would not refer to MoPOC? Tell me about those patients.

**Tell me how MoPOC has affected the care provided to patients**.

*(RE-AIM; Effectiveness)*

- What impact, if any, has it had on:
  - Quality of care provided?
  - Scope of care provided?
  - Coordination of care?
  - Access to care?
  - *If needed*: Tell me about a patient for whom MoPOC has made a difference.

**How is/isn’t MoPOC:**

*(RE-AIM; Reach, Effectiveness, Adoption, Implementation)*

- Reaching the right patients?
- Providing the right services?
- Providing services in the right locations?

What, if anything, should MoPOC change?

**What challenges, if any, are there to keeping MoPOC going at your site long term?**

*(RE-AIM; Maintenance)*

If needed: What could get in the way of keeping MoPOC up and running?

If needed: What plans, if any, have been made to continue MoPOC after ORH funding is over?

## Wrap Up Questions

What else should VA know about MoPOC at your site?

What suggestions do you have to improve MoPOC?

If another site was considering rolling out MoPOC, what advice would you give them?

Given your experience so far, what would you recommend to VA about adopting MoPOC?

Who else could help us understand your/your site’s experience with MoPOC so far?
